# Levetiracetam vs. Fosphenytoin for Second-Line Treatment of Status Epilepticus: Propensity Score Matching Analysis Using a Nationwide Inpatient Database

**DOI:** 10.3389/fneur.2020.00615

**Published:** 2020-07-02

**Authors:** Kensuke Nakamura, Hiroyuki Ohbe, Hiroki Matsui, Yuji Takahashi, Aiki Marushima, Yoshiaki Inoue, Kiyohide Fushimi, Hideo Yasunaga

**Affiliations:** ^1^Department of Emergency and Critical Care Medicine, Hitachi General Hospital, Hitachi, Japan; ^2^Department of Clinical Epidemiology and Health Economics, School of Public Health, The University of Tokyo, Tokyo, Japan; ^3^Department of Emergency and Critical Care Medicine, Tsukuba University Hospital, Tsukuba, Japan; ^4^Department of Health Policy and Informatics, Graduate School of Medicine, Tokyo Medical and Dental University, Tokyo, Japan

**Keywords:** epilepsy, fosphenytoin, phenytoin, levetiracetam, seizure

## Abstract

**Objective:** Status epilepticus is a major emergency condition. The choice of antiepileptic drugs for second-line treatment after benzodiazepine remains controversial, including levetiracetam vs. fosphenytoin. We compare the safety of intravenous levetiracetam and fosphenytoin as a second-line treatment in patients with status epilepticus using a nationwide database.

**Methods:** An observational study conducted with the Japanese Diagnosis Procedure Combination inpatient database identified adult patients who had been admitted for status epilepticus and who had received intravenous diazepam on the day of admission from March 1, 2011 to March 31, 2018. Patients who received intravenous levetiracetam on the day of admission were defined as the levetiracetam group and those who received intravenous fosphenytoin on the day of admission were defined as the fosphenytoin group. Propensity score matching was performed to compare outcomes obtained for the levetiracetam and fosphenytoin groups.

**Results:** The analysis examined data of 5,667 patients. Overall, 1,403 (25%) patients received levetiracetam; 4,264 (75%) received fosphenytoin. One-to-one propensity score matching created 1,363 matched pairs. No significant difference was found in in-hospital mortality (5.2 vs. 5.1%; odds ratio, 1.03; 95% confidence interval, 0.73–1.46). The proportion of vasopressor use on the day of admission was significantly lower for the levetiracetam group than for the fosphenytoin group (3.2 vs. 4.9%; odds ratio, 0.63; 95% confidence interval, 0.43–0.92). No significant difference was found in other secondary outcomes including total hospitalization cost.

**Conclusion:** Levetiracetam was related to significantly reduced vasopressor use on the day of admission than that found for fosphenytoin, in adult status epilepticus.

## Introduction

Status epilepticus (SE) is a major medical emergency condition, and failure to treat SE would cause death or irreversible cerebral damage (1, 2). Benzodiazepines are used as first-line treatment for SE (3, 4). Second-line treatments of longer-acting antiepileptic drugs (AEDs) are administered to prevent recurrence (5).

The choice of AEDs for second-line treatment remains controversial. Phenytoin and fosphenytoin have been used as second-line treatment (6). Levetiracetam, a new AED, binds to the synaptic vesicle protein 2A and regulates the release of neurotransmitters. Earlier observational studies show that levetiracetam is similarly effective and that it is associated with less adverse effects than those of phenytoin (7–11). Similar findings have been reported from small randomized control studies (12–14). Neurocritical Care Society guidelines recommended levetiracetam use in addition to phenytoin/fosphenytoin (5), but other guidelines do not recommend levetiracetam use (15, 16).

Fosphenytoin reportedly offers several potential advantages over phenytoin (17). However, evidence for the efficacy and safety of fosphenytoin compared with levetiracetam as second-line treatment for SE is sparse. Only one small retrospective study (*n* = 63) has shown that the efficacies of both drugs might be equivalent. Blood pressure reduction was observed in two cases in fosphenytoin group but was not observed in the levetiracetam group (8). No reported randomized control trial has compared levetiracetam to fosphenytoin for SE.

Therefore, the present study uses a nationwide inpatient database in Japan to compare the safety of intravenous levetiracetam and intravenous fosphenytoin as a second-line treatment in patients with SE. Furthermore, we evaluate the efficacy by comparing the use of third-line AEDs as surrogate outcomes of seizure cessation.

## Materials and Methods

### Data Source

The study was designed as an observational study using routinely collected data. We used the Japanese Diagnosis Procedure Combination inpatient database, which includes discharge abstracts and administrative claims data from more than 1,200 acute-care hospitals. It covers ~90% of all tertiary-care emergency hospitals in Japan. The database includes the following data: age; sex; smoking history; body weight; body height; level of consciousness at admission; diagnoses (main diagnosis, comorbidities present at admission, and conditions arising after admission) recorded according to the International Classification of Diseases Tenth Revision (ICD-10) codes and written in Japanese text; procedures; prescriptions; drug administration; and discharge status. Because the diagnostic records are linked to a payment system, attending physicians must report objective evidence for their diagnoses for purposes of treatment cost reimbursement (18). An earlier study of records of diagnoses and procedures in the database established their validity (19). The specificity of diagnoses exceeded 96%, whereas the sensitivity was 50–80%. The specificity and sensitivity of procedures each exceeded 90% (19).

### Study Population

We identified all patients with emergency admission for SE (ICD-10 code: G41) and discharged from March 1, 2011 through March 31, 2018 (20). We did not include patients who developed SE after admission. We excluded the following patients: (i) younger than 15 years of age; (ii) pregnant; (iii) at the second or subsequent admission with a diagnosis of SE during the study period; (iv) planned admission; (v) admitted with epilepsy mimickers (ICD-10 codes: F41, F44, F51, G43, G45, G47, H81, R55); (vi) admitted with out-of-hospital cardiac arrest (ICD-10 code: F46); (vii) those who did not receive intravenous diazepam on the day of admission; (viii) those who neither received intravenous levetiracetam nor intravenous fosphenytoin on the day of admission; and (x) those who received both intravenous levetiracetam and intravenous fosphenytoin on the day of admission.

### Group Assignment

Patients who received intravenous levetiracetam on the day of admission were defined as the levetiracetam group. Those who received intravenous fosphenytoin on the day of admission were defined as the fosphenytoin group.

### Covariates and Outcomes

Covariates included age, sex, smoking history (non-smoker, current/past smoker, unknown), body mass index at admission, Japan Coma Scale at admission (21), Charlson comorbidity index (22), type of SE (20) etiology of SE, ambulance use, visiting holiday or night hours by ambulance, teaching hospital, examination on the day of admission (computed tomography, magnetic resonance imaging, cerebrospinal fluid analysis, and electroencephalogram), intravenous AEDs on the day of admission (50% glucose, vitamin B1, phenytoin, phenobarbital, midazolam, thiamylal, thiopental, and propofol) (23), and numbers of intravenous AEDs on the day of admission.

The body mass index was categorized as <18.5, 18.5–24.9, 25.0–29.9, or ≥30.0 kg/m^2^, or missing data. Japan Coma Scale status was categorized as alert, confusion, somnolence, and coma. Japan Coma Scale status has been shown to be well-correlated with Glasgow Coma Scale score (21). The Charlson comorbidity index, which was scored using diagnoses for individual patients, was 0, 1, 2, or ≥3. Type of SE was categorized as tonic–clonic SE (ICD-10 code: G410), epileptic absence status (G411), complex partial SE (G412), or others and unspecified (G418, G419) (20). Etiology of SE was defined using the ICD-10 diagnosis codes at admission given in Supplementary Table 1.

The primary outcome was in-hospital mortality. Secondary outcomes were death within 24 h, length of hospital stay, total hospitalization cost, Japan Coma Scale at discharge, mechanical ventilation on the day of admission, and vasopressor use on the day of admission.

### Statistical Analysis

A propensity score matching method was applied to compare outcomes between levetiracetam and fosphenytoin groups (24, 25). A multivariable logistic regression model was employed to predict the propensity scores of the patients receiving intravenous levetiracetam on the day of admission, using all the covariates presented in Table 1 as predictor variables. One-to-one nearest-neighbor matching without replacement was then performed for the estimated propensity scores of the patients using a caliper width set at 20% of the standard deviation for the propensity scores (24, 25). To assess the performance of the matching, the covariates before and after propensity score matching were compared using absolute standardized differences (26). In this evaluation, absolute standardized differences ≤ 10% were regarded as denoting negligible imbalances between the levetiracetam and fosphenytoin groups (26). We conducted propensity score matching using the STATA module of PSMATCH2 software provided by Leuven and Sianesi (27).

**Table 1:** Baseline characteristics before and after propensity score matching.

	**Overall cohort**			**Matched cohort**		
	**LEV**	**FPHT**		**LEV**	**FPHT**	
**Covariates**	**(*n* = 1,403)**	**(*n* = 4,264)**	**ASD**	**(*n* = 1,363)**	**(*n* = 1,363)**	**ASD**
Age, yr, mean (SD)	67 (18)	64 (19)	15.2	67 (18)	67 (18)	1.2
Male, *n* (%)	819 (58.4)	2,508 (58.8)	0.9	794 (58.3)	830 (60.9)	5.4
Smoking history, *n* (%)						
Non-smoker	873 (62.2)	2,550 (59.8)	5.0	843 (61.8)	858 (62.9)	2.3
Current/past smoker	311 (22.2)	1,041 (24.4)	5.3	305 (22.4)	323 (23.7)	3.1
Unknown	219 (15.6)	673 (15.8)	0.5	215 (15.8)	182 (13.4)	6.9
Body mass index, kg/m^2^, *n* (%)						
<18.5	267 (19.0)	790 (18.5)	1.3	257 (18.9)	233 (17.1)	4.6
18.5–24.9	741 (52.8)	2,236 (52.4)	0.8	721 (52.9)	742 (54.4)	3.1
25.0–29.9	167 (11.9)	516 (12.1)	0.6	160 (11.7)	171 (12.5)	2.5
≥30.0	36 (2.6)	126 (3.0)	2.4	35 (2.6)	31 (2.3)	1.9
Missing	192 (13.7)	596 (14.0)	0.8	190 (13.9)	186 (13.6)	0.9
Japan Coma Scale at admission, *n* (%)						
Alert	134 (9.6)	449 (10.5)	3.3	130 (9.5)	128 (9.4)	0.5
Confusion	419 (29.9)	1,226 (28.8)	2.4	409 (30.0)	400 (29.3)	1.4
Somnolence	255 (18.2)	803 (18.8)	1.7	251 (18.4)	257 (18.9)	1.1
Coma	595 (42.4)	1,786 (41.9)	1.1	573 (42.0)	578 (42.4)	0.7
Charlson comorbidity index, *n* (%)						
0	674 (48.0)	2,126 (49.9)	3.6	659 (48.3)	652 (47.8)	1.0
1	392 (27.9)	1,222 (28.7)	1.6	376 (27.6)	373 (27.4)	0.5
2	191 (13.6)	536 (12.6)	3.1	185 (13.6)	199 (14.6)	3.0
≥3	146 (10.4)	380 (8.9)	5.1	143 (10.5)	139 (10.2)	1.0
Type of status epilepticus, *n* (%)						
Tonic–clonic status epilepticus	141 (10.0)	497 (11.7)	5.2	140 (10.3)	135 (9.9)	1.2
Epileptic absence status	15 (1.1)	30 (0.7)	3.9	14 (1.0)	14 (1.0)	0.0
Complex partial status epilepticus	93 (6.6)	229 (5.4)	5.3	89 (6.5)	93 (6.8)	1.2
Others or unspecified	1,154 (82.3)	3,508 (82.3)	0.0	1,120 (82.2)	1,121 (82.2)	0.2
Etiology of status epilepticus, *n* (%)						
Brain neoplasm	45 (3.2)	176 (4.1)	4.9	44 (3.2)	45 (3.3)	0.4
Subarachnoid or intracerebral hemorrhage	121 (8.6)	383 (9.0)	1.3	116 (8.5)	112 (8.2)	1.1
Cerebral infarction	249 (17.7)	643 (15.1)	7.2	242 (17.8)	221 (16.2)	4.1
Other cerebral vascular etiologies	51 (3.6)	139 (3.3)	2.1	49 (3.6)	49 (3.6)	0.0
Traumatic brain injury	63 (4.5)	145 (3.4)	5.6	59 (4.3)	51 (3.7)	3.0
Inflammation/immune etiologies	12 (0.9)	30 (0.7)	1.7	12 (0.9)	7 (0.5)	4.4
Neurodegenerative etiologies	91 (6.5)	238 (5.6)	3.8	90 (6.6)	78 (5.7)	3.7
Metabolic etiologies	58 (4.1)	145 (3.4)	3.9	56 (4.1)	50 (3.7)	2.3
Brain infections	20 (1.4)	34 (0.8)	6.0	19 (1.4)	20 (1.5)	0.6
Intoxication	4 (0.3)	16 (0.4)	1.6	4 (0.3)	2 (0.1)	3.1
Other etiologies	45 (3.2)	170 (4.0)	4.2	45 (3.3)	42 (3.1)	1.3
Undetermined	758 (54.0)	2,422 (56.8)	5.6	737 (54.1)	783 (57.4)	6.8
Ambulance use, *n* (%)	1,245 (88.7)	3,762 (88.2)	1.6	1,207 (88.6)	1,202 (88.2)	1.1
Visiting holiday or night hours by ambulance, *n* (%)	205 (14.6)	532 (12.5)	6.2	198 (14.5)	202 (14.8)	0.8
Teaching hospital, *n* (%)	1,129 (80.5)	3,668 (86.0)	14.9	1,106 (81.1)	1,117 (82.0)	2.1
Examinations on the day of admission, *n* (%)						
Computed tomography	1,206 (86.0)	3,769 (88.4)	7.3	1,171 (85.9)	1,169 (85.8)	0.4
Magnetic resonance imaging	581 (41.4)	1,545 (36.2)	10.6	566 (41.5)	568 (41.7)	0.3
Cerebrospinal fluid analysis	128 (9.1)	383 (9.0)	0.5	122 (9.0)	119 (8.7)	0.8
Electroencephalogram	187 (13.3)	582 (13.6)	0.9	180 (13.2)	171 (12.5)	2.0
Intravenous antiepileptic drugs on the day of admission, *n* (%)						
50% glucose	34 (2.4)	104 (2.4)	0.1	33 (2.4)	23 (1.7)	5.2
Vitamin B1	44 (3.1)	136 (3.2)	0.3	44 (3.2)	41 (3.0)	1.3
Phenytoin	150 (10.7)	135 (3.2)	30.0	111 (8.1)	121 (8.9)	2.6
Phenobarbital	100 (7.1)	211 (4.9)	9.2	94 (6.9)	93 (6.8)	0.3
Midazolam	222 (15.8)	641 (15.0)	2.2	208 (15.3)	222 (16.3)	2.8
Thiamylal	3 (0.2)	19 (0.4)	4.0	2 (0.1)	2 (0.1)	0.0
Thiopental	4 (0.3)	9 (0.2)	1.5	4 (0.3)	3 (0.2)	1.4
Propofol	134 (9.6)	413 (9.7)	0.5	129 (9.5)	116 (8.5)	3.3
Number of intravenous antiepileptic drugs on the day of admission						
1	904 (64.4)	3,051 (71.6)	15.3	904 (66.3)	892 (65.4)	1.9
2	398 (28.4)	1,010 (23.7)	10.7	378 (27.7)	391 (28.7)	2.1
≥3	101 (7.2)	203 (4.8)	10.3	81 (5.9)	80 (5.9)	0.3

*Baseline characteristics of the status epilepticus included in this study are listed. There is no significant difference between levetiracetam and fosphenytoin group, in overall cohort and matched cohort. SD, standard deviation; LEV, levetiracetum; FPHT, fosphenytoin; ASD, absolute standardized difference*.

We used a generalized estimating equation approach for comparisons of the primary and secondary outcomes, accompanied by cluster-robust standard errors with hospitals used as the cluster variable (28). Odds ratios and their 95% confidence intervals were calculated for binary outcomes and β coefficients. Their 95% confidence intervals were calculated for the continuous outcomes. These estimates were obtained using generalized estimating equation regression models with logit link function for odds ratios and identity link function for β coefficients.

We conducted two sensitivity analyses. First, to confirm the robustness of the main result by application of a different model, we performed a propensity score adjustment analysis. For this sensitivity analysis, we performed a multivariable regression model with generalized estimation equations accompanied by cluster-robust standard errors with hospitals used as the cluster variable in the overall cohort. Each primary and secondary outcome was defined as the dependent variable and the intravenous levetiracetam on the day of admission. The estimated propensity scores from the main analyses were used for covariates. Second, to compare the effects of seizure cessation rates of the levetiracetam and fosphenytoin groups, the use of third-line treatments of AEDs on the day of admission were compared as surrogate outcomes for the seizure cessation rate between the two groups. We defined midazolam, thiamylal, thiopental, and propofol as third-line treatments according to Japanese epilepsy guidelines (29). For this sensitivity analysis, we calculated the propensity scores of receiving levetiracetam on the day of admission, using the covariates presented in Table 1 except third-line treatments of AEDs and the number of intravenous AEDs on the day of admission. Then, we applied propensity score matching analysis again using these propensity scores and compared the use of third-line treatments of AEDs between two groups using a generalized estimating equation approach.

Categorical variables were described as a number and percentage. Continuous variables were presented as the mean and standard deviation. All reported *p*-value were two-sided; values for which *p* <0.05 were inferred as significant. All analyses were conducted using software (STATA/MP 15.0; Stata Corp. College Station, TX, USA).

## Results

**Figure 1:**
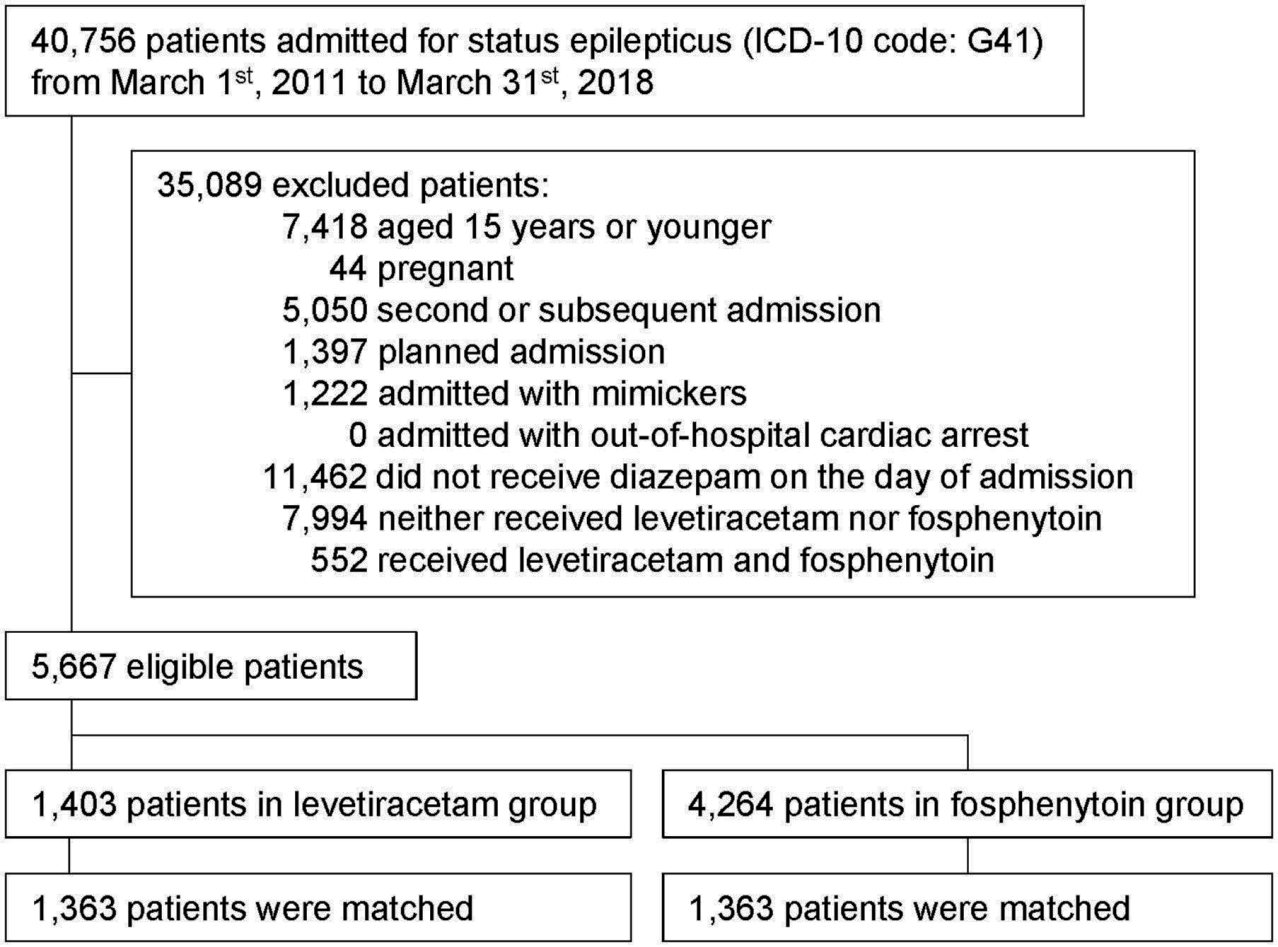
Study outline. Patient flowchart is shown. Forty thousand seven hundred and fifty-six patients with status epilepticus were extracted within the study period. After 35,089 exclusion, 5,667 eligible patients were included into this study. Then, we performed propensity score matching for levetiracetam and fosphenytoin group, and finally 1,363 patients in each group is analyzed.

This study examined data of 5,667 eligible patients (Figure 1). Of these, 1,403 patients were assigned to the levetiracetam group (received intravenous levetiracetam on the day of admission); 4,264 patients were assigned to the fosphenytoin group (received intravenous fosphenytoin on the day of admission). Among the levetiracetam group, intravenous levetiracetam was administered at a dose of mean 870 ± standard deviation 554 mg and median 500 mg (interquartile range 500–1,000 mg). Among the fosphenytoin group, intravenous fosphenytoin was administered at a dose of mean 1,207 ± standard deviation 495 mg and median 1,350 mg (interquartile range 750–1,500 mg).

Table 1 presents characteristics of patients in two groups before and after propensity score matching. Most patients were elderly and were transported by ambulance. One-to-one propensity score matching created 1,363 matched pairs. After propensity score matching, the patient characteristics were well-balanced between the two groups.

Table 2 shows outcomes obtained before and after propensity score matching. Before propensity score matching, the overall in-hospital mortality was 4.7% (267/5,667). After propensity score matching, no significant difference was found in in-hospital mortality (5.2 vs. 5.1%; odds ratio, 1.03; 95% confidence interval: 0.73, 1.46). No significant difference was found between two groups in secondary outcomes including total hospitalization cost, except vasopressor use on the day of admission (3.2 vs. 4.9%; odds ratio, 0.63; 95% confidence interval, 0.43–0.92).

**Table 2:** Outcomes in overall and matched cohorts and propensity score matching analysis results.

	**Unmatched cohort**		**Matched cohort**			
	**LEV**	**FPHT**	**LEV**	**FPHT**	**Odds ratios or β**	
**Outcomes**	**(*n* = 1,403)**	**(*n* = 4,264)**	**(*n* = 1,363)**	**(*n* = 1,363)**	**Coefficient (95% CI)**	***P*-value**
In-hospital mortality, *n* (%)	71 (5.1)	196 (4.6)	71 (5.2)	69 (5.1)	1.03 (0.73, 1.46)	0.87
Death within 24 h	1 (0.1)	16 (0.4)	1 (0.1)	5 (0.4)	0.20 (0.02, 1.71)	0.14
Length of hospital stay, days, mean (SD)	22 (26)	23 (31)	22 (26)	24 (33)	−1.72 (−4.13, 0.69)	0.16
Total hospitalization cost, USD, mean (SD)	9,615 (10,596)	9,691 (11,907)	9,516 (10,523)	9,835 (11,950)	−358 (−1,286, 569)	0.45
Japan Coma Scale at discharge, *n* (%)						
Alert	773 (55)	2,421 (57)	754 (55)	734 (54)	1.06 (0.91, 1.23)	0.43
Confusion	498 (36)	1,432 (34)	480 (35)	499 (37)	0.94 (0.80, 1.12)	0.51
Somnolence	44 (3.1)	147 (3.4)	43 (3.2)	46 (3.4)	0.98 (0.64, 1.50)	0.91
Coma	17 (1.2)	68 (1.6)	15 (1.1)	15 (1.1)	0.96 (0.47, 1.95)	0.90
Mechanical ventilation on the day of admission	184 (13)	600 (14)	174 (13)	187 (14)	0.89 (0.70, 1.14)	0.35
Vasopressor use on the day of admission	46 (3.3)	183 (4.3)	43 (3.2)	67 (4.9)	0.63 (0.43, 0.92)	0.017

*The outcomes in unmatched and matched cohort are shown. There is no significant difference in mortality, length of hospital stay, hospitalization cost, consciousness disturbance at discharge and mechanical ventilation between levetiracetam and fosphenytoin group. However, vasopressor use on the day of admission was significantly lower in levetiracetam group than in fosphenytoin group. SD, standard deviation; LEV, levetiracetum; FPHT, fosphenytoin; CI, confidence interval; USD, United States dollars*.

Results of sensitivity analyses for propensity score adjustment analysis were consistent with results obtained from the main analysis (Table 3).

**Table 3:** Results of sensitivity analysis for propensity score adjustment analysis in the overall cohort.

	**LEV**	**FPHT**	**Odds ration or β coefficient**	
**Outcomes**	**(*n* = 1,403)**	**(*n* = 4,264)**	**(95% CI)**	***P*-value**
In hospital mortality, *n* (%)	71 (5.1)	196 (4.6)	1.00 (0.75, 1.34)	0.99
Death within 24 h	1 (0.1)	16 (0.4)	0.19 (0.02, 1.48)	0.11
Length of hospital stay, days, mean (SD)	22 (26)	23 (31)	−1.94 (−3.97, 0.09)	0.061
Total hospitalization cost, USD, mean (SD)	9,615 (10,596)	9,691 (11,907)	−492 (−1,274, 290)	0.22
Japan Coma Scale at discharge, *n* (%)				
Alert	773 (55)	2,421 (57)	1.00 (0.88, 1.14)	0.96
Confusion	498 (36)	1,432 (34)	1.01 (0.88, 1.17)	0.88
Somnolence	44 (3.1)	147 (3.4)	0.84 (0.58, 1.20)	0.34
Coma	17 (1.2)	68 (1.6)	0.71 (0.42, 1.22)	0.21
Mechanical ventilation on the day of admission	184 (13)	600 (14)	0.93 (0.77, 1.14)	0.51
Vasopressor use on the day of admission	46 (3.3)	183 (4.3)	0.69 (0.50, 0.97)	0.034

*The outcomes in the overall cohort are shown. Results of sensitivity analyses is consistent with results obtained from the main analysis. SD, standard deviation; LEV, levetiracetum; FPHT, fosphenytoin; CI, confidence interval; USD, United States dollars*.

Results of sensitivity analysis for comparison of the use of third-line treatments of AEDs on the day of admission as surrogate outcome for the seizure cessation rate show no significant difference between two groups in the use of third-line treatments of AEDs (22.4 vs. 21.9%; odds ratio, 1.01; 95% confidence interval, 0.82–1.24).

## Discussion

This nationwide observational study compared the effects of intravenous levetiracetam and intravenous fosphenytoin as second-line treatment in patients with SE. After adjustment for covariates, in-hospital mortality was found to be similar in both groups, but vasopressor use on the day of admission was significantly lower in the levetiracetam group than in the fosphenytoin group. Furthermore, results obtained from the use of third-line treatments of AED, as surrogate outcome of seizure cessation, were not different between the levetiracetam and fosphenytoin group in sensitivity analyses.

Outcomes including in-hospital mortality, death within 24 h, neurological outcome at discharge, mechanical ventilation and the use of third-line treatments on the day of admission were not significantly different between the levetiracetam and fosphenytoin groups examined in this study. These findings were consistent with those of earlier RCTs comparing levetiracetam and phenytoin (12–14) and earlier observational studies comparing levetiracetam and phenytoin or fosphenytoin (8–11, 30).

In terms of the adverse events, our results suggest that levetiracetam is superior to fosphenytoin in vasopressor use on the day of admission. Earlier studies showed that severe adverse events such as cardiac arrest or hypotension occurred in the phenytoin group, but not in the levetiracetam group (12–14). However, because the sample size was insufficient to detect such a small difference in the adverse events, no significant difference was found in adverse effects in earlier studies. One major strength of the current study is the larger sample size than those of earlier studies. Using the nationwide population, this study first showed the significant difference of adverse events between levetiracetam and fosphenytoin. The circulation failure in SE was crucially important not only for life-saving but also for protecting the brain from neuronal injury (31). Because subsequent cognitive dysfunctions would often be associated with SE (32, 33), maintaining circulation in patients with SE is exceedingly important. Therefore, AEDs that can induce circulatory failure with even slight frequency should be avoided.

Phenytoin, as a sodium channel blocker, potentially entails cardiovascular adverse effects including arrhythmia and hypotension (34, 35). Fosphenytoin is the pro-drug of phenytoin; its side effects were designed to be reduced. Nevertheless, fosphenytoin has side effects of blood pressure reduction and arrhythmia (17). Moreover, anticonvulsant hypersensitivity syndrome, which presents fever, rash, and liver injury, is associated primarily with phenytoin/fosphenytoin administration (36). The main mechanism of levetiracetam is regarded as binding to the synaptic vesicle protein 2A and regulating the release of neurotransmitters, although not all mechanisms have been clarified (7, 37). Levetiracetam has no direct effect on the naive gamma amino butyric acid synapses or sodium channel. Therefore, levetiracetam is regarded as having weaker systemic side effects than other AEDs (37, 38). The plasma half-life is 6–8 h, with <10% protein binding. The metabolic pathway is an enzymatic hydrolysis of the acetamide group, which has less drug interaction. The metabolites have no pharmacological activity. They are excreted renally without hepatic metabolism (39). These pathophysiological and pharmacokinetic mechanisms can support our results by virtue of reduced side effects in the levetiracetam group compared to those of the fosphenytoin group.

This study has some limitations. First, the diagnosis of SE based on diagnostic codes has not been well-validated in the Japanese Diagnosis Procedure Combination inpatient database. However, an earlier validation study conducted using another database showed excellent specificity from the combination of ICD-10 code and the use of AEDs (40). Second, the ICD-10 codes for the type of SE were under-recorded. Most of the patients were classified as others or as unspecified. Because most of the included patients were admitted by ambulance in this study and because such patients would be tonic-clonic SE in Japan (8), the mos others or unspecified might be patients with tonic-clonic SE. Third, the observational characteristics of the study design leave it open to potential bias and confounding. We attempted to control this confounding using propensity score analyses, but we were unable to control for possible unmeasured variables. Fourth, the mean levetiracetam dosage used for this study was lower than those recommended in the guidelines (5) or those in previous randomized control trials (41, 42). The lower levetiracetam dosage in our study might be attributable to the fact that the maximum dose of levetiracetam is 3,000 mg/day in Japanese national health insurance coverage. The lower dose of levetiracetam might be associated with reduced vasopressor use in exchange for unsuccess of seizure cessation. However, results of our sensitivity analyses showed no significant difference in the use of third-line treatments of AED as a surrogate outcome for the seizure cessation rate. Finally, the seizure cessation rate and the time from drug administration to the seizure cessation should be evaluated primarily as the efficacy of SE. Nevertheless, this point could not be analyzed in this study.

## Conclusions

Results of this large nationwide observational study demonstrated that the intravenous levetiracetam is associated with reduced vasopressor use on the day of admission compared with intravenous fosphenytoin for the second line treatment after diazepam of adult patients with SE.

## Data Availability Statement

The datasets generated for this study are available on request to the corresponding author.

## Ethics Statement

The studies involving human participants were reviewed and approved by the Institutional Review Board of The University of Tokyo. Written informed consent from the participants' legal guardian/next of kin was not required to participate in this study in accordance with the national legislation and the institutional requirements.

## Author Contributions

KN: conception, conduction of the study, and drafting the manuscript. HO: data analysis, interpretation, and drafting of the manuscript. HM: data analysis. YT, AM, and YI: interpretation and contribution to the manuscript. KF: revision of the manuscript. HY: revision of the manuscript and supervision of the study. All authors: read and approved the manuscript.

## Conflict of Interest

The authors declare that the research was conducted in the absence of any commercial or financial relationships that could be construed as a potential conflict of interest.
