# Supplementary material for: Levetiracetam vs. Fosphenytoin for Second-Line Treatment of Status Epilepticus: Propensity Score Matching Analysis Using a Nationwide Inpatient Database
Source: Front Neurol. 2020 Jul 2;11:615. doi: 10.3389/fneur.2020.00615 (PMC7348044; doi:10.3389/fneur.2020.00615)
Supplement: Supplementary file 1 [file Table_1.DOCX]

**Supplementary Table 1** ICD-10 codes for etiology of status epilepticus

| ICD-10 codes |
| --- |
| **Subarachnoid or intracerebral haemorrhage**  I60, I61, I690, I691  **Cerebral infarction**  I63, I693  **Other cerebral vascular etiologies**  G08, G46, I62, I64, I67, I68, I694, I698, Q282, Q283  **Metabolic etiologies**  E035, E05, E100, E101, E110, E111, E120, E121, E130, E131, E140, E141, E15, E161, E162, E222, E232, E51, E52, E53, E61, E70, E71, E72, E73, E74, E75, E76, E77, E80, E83, E870, E871  **Intoxication**  G92, T36, T37, T38, T39, T4, T5, T60, T61, T62, T63, T64, T65, T96, T97  **Traumatic brain injury**  S06, T790, T791, T905  **Brain neoplasm**  C70, C71, C793, D430, D431, D432, D439, D33  **Inflammation/immune etiologies**  G03, G040, G041, G048, G049, G058  **Neurodegenerative etiologies**  G10, G12, G13, G20, G21, G22, G23, G30, G31, G32, G35, G36, G37  **Brain infections**  G00, G01, G02, G042, G050, G051, G052, G060, G062, G07  **Other etiologies**  G09, G80, G91, G930, G931, G932, G933, G934, G935, G936, G937, G939, G94, I46, P20, P21, Q0, T670, T671, T672, T703  **Undetermined** |
| None of above |

ICD-10, International Classification of Diseases, Tenth Revision
